# Environmental Predictors of Seasonal Influenza Epidemics across Temperate and Tropical Climates

**DOI:** 10.1371/journal.ppat.1003194

**Published:** 2013-03-07

**Authors:** James D. Tamerius, Jeffrey Shaman, Wladmir J. Alonso, Kimberly Bloom-Feshbach, Christopher K. Uejio, Andrew Comrie, Cécile Viboud

**Affiliations:** 1 Environmental Health Sciences, Columbia University, New York, New York, United States of America; 2 Fogarty International Center, National Institutes of Health, Bethesda, Maryland, United States of America; 3 Mount Sinai School of Medicine, New York, New York, United States of America; 4 Department of Geography, Florida State University, Tallahassee, Florida, United States of America; 5 School of Geography and Development, University of Arizona, Tucson, Arizona, United States of America; Imperial College London, United Kingdom

## Abstract

Human influenza infections exhibit a strong seasonal cycle in temperate regions. Recent laboratory and epidemiological evidence suggests that low specific humidity conditions facilitate the airborne survival and transmission of the influenza virus in temperate regions, resulting in annual winter epidemics. However, this relationship is unlikely to account for the epidemiology of influenza in tropical and subtropical regions where epidemics often occur during the rainy season or transmit year-round without a well-defined season. We assessed the role of specific humidity and other local climatic variables on influenza virus seasonality by modeling epidemiological and climatic information from 78 study sites sampled globally. We substantiated that there are two types of environmental conditions associated with seasonal influenza epidemics: “cold-dry” and “humid-rainy”. For sites where monthly average specific humidity or temperature decreases below thresholds of approximately 11–12 g/kg and 18–21°C during the year, influenza activity peaks during the cold-dry season (i.e., winter) when specific humidity and temperature are at minimal levels. For sites where specific humidity and temperature do not decrease below these thresholds, seasonal influenza activity is more likely to peak in months when average precipitation totals are maximal and greater than 150 mm per month. These findings provide a simple climate-based model rooted in empirical data that accounts for the diversity of seasonal influenza patterns observed across temperate, subtropical and tropical climates.

## Author Summary

Human influenza infections have a pronounced seasonal cycle in temperate regions. Recent laboratory and epidemiological evidence suggests that low humidity conditions in the winter may increase virus survival and enable the virus to transmit efficiently between hosts. However, seasonal influenza activity in some tropical locations occurs during the rainy season, whereas other tropical locations do not experience a well-defined influenza season. The primary goal of this study was to identify the relationship between the timing of seasonal influenza epidemics and climate variability across the globe. We show the importance of thresholds in humidity, temperature and precipitation that affect the epidemiology, and potentially the transmission route, of influenza. A better understanding of the environmental, demographic and behavioral drivers of influenza seasonality is important for optimizing intervention strategies, especially in low and middle-latitude regions.

## Introduction

Influenza exerts a significant health burden on human populations across temperate, subtropical and tropical regions [1]. The striking seasonal pattern that characterizes influenza in temperate populations has long suggested a causal link between seasonal fluctuations in climatic and social factors and influenza transmission [2]–[4]. Temperate regions of the northern and southern hemispheres are characterized by highly synchronized annual influenza epidemics during their respective winter months [5], [6]. In contrast, influenza seasonal characteristics are more diverse in tropical and subtropical regions; some sites experience annual epidemics coinciding with the local rainy season [7]–[11], whereas others are characterized by semi-annual epidemics or year-round influenza activity without well-defined influenza seasons [7], [12], [13].

Recent epidemiological studies indicate that low levels of specific humidity are associated with the onset of pandemic and epidemic influenza in the US [14], [15], consistent with laboratory experiments and animal models suggesting that low specific humidity favors virus survival and aerosol transmission [16]–[17]. There are several alternative explanations for the winter seasonal transmission of influenza in temperate regions, including the inhibition of host immune function due to decreased exposure to solar radiation [18], [19], and the inhibition of mucociliary clearance by the inhalation of cold-dry air [20]. Person-to-person contact rates may also strengthen in the winter due to increased indoor crowding, modulated by school terms [21]. There are few biological explanations for the association between precipitations and influenza activity reported in some tropical and subtropical regions, although rainy conditions may also favor indoor crowding [4].

Although it is common for epidemiological studies to examine relationships between seasonal influenza activity and climatic factors for individual sites, few studies have assessed the consistency of these relationships across a broad range of temperate, subtropical and tropical sites. A recent study evidenced a link between influenza and temperature based on aggregate country-level data, but did not characterize the geographical and climatic boundaries that define regions experiencing different influenza seasonality patterns [22]. Here we investigate both relative and absolute associations between climatic factors and the timing of seasonal influenza epidemics for 78 individual sites sampled globally [23]. We develop models that predict the month of peak influenza activity for each study site as a function of climatic variables and identify climatic thresholds accounting for the diversity of influenza seasonality patterns observed globally.

## Methods

### Data

#### Influenza epidemiological data

We used a recently developed global database that provides information on the month of maximum influenza activity (“influenza peaks”) for 78 sites worldwide, of which 39% were located in the tropics. A detailed description of the database is provided in the Supplement and [23], and is briefly summarized below.

The data were compiled based on a systematic literature review of published influenza and respiratory virus surveillance studies, reporting weekly or monthly laboratory-confirmed influenza cases for a period of 12 consecutive months or more, augmented with electronic data from regional or national influenza surveillance schemes. Studies focused on the 2009 A/H1N1 pandemic virus were excluded to restrict the analysis to seasonal patterns of inter-pandemic influenza.

**Figure 1:**
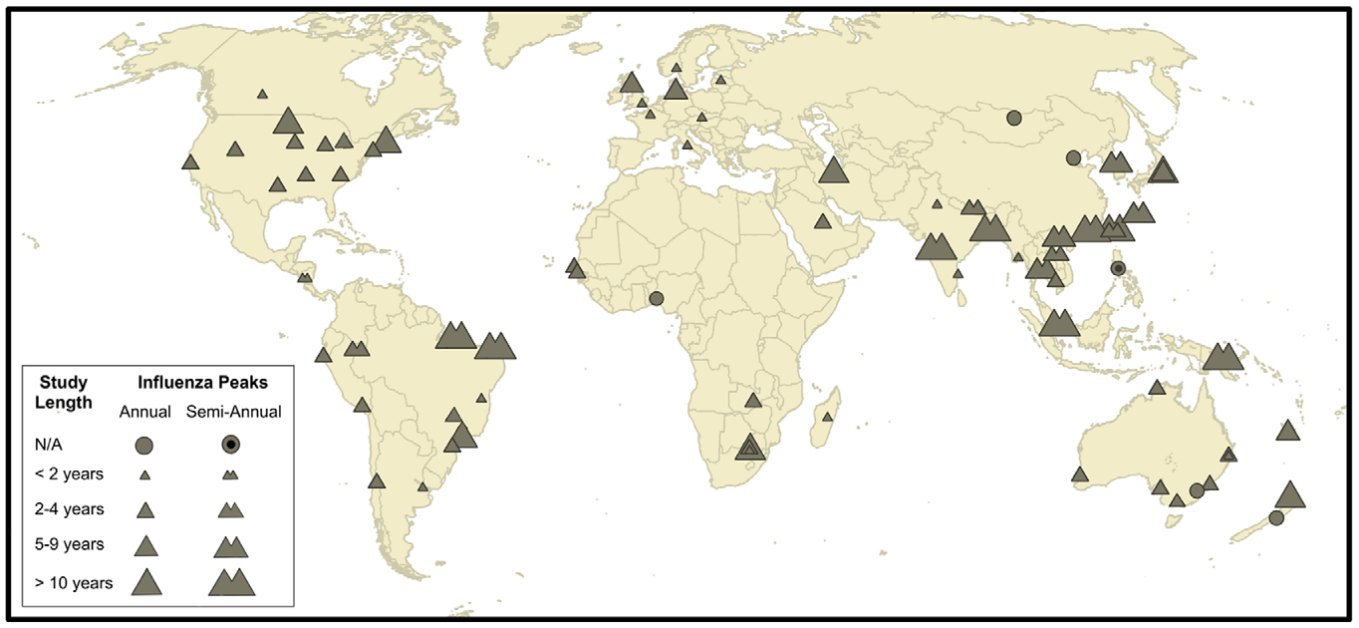
Map of 78 study sites included in this study. The site symbols indicate whether a location has annual or semi-annual influenza activity, and symbol size is proportional to the duration of the epidemiological studies used to determine the month of peak activity for each location.

We identified 85 studies matching our inclusion criteria, encompassing 78 sites in 40 countries sampled during the period 1975–2008, with median study duration of 2 years (Figure 1). A majority of study sites (76%) represented a specific city rather than a state, province, or region. The 24% of study sites representing regional level data encompassed areas that were relatively small and homogeneous with respect to climate, including countries such as Italy and the Republic of Korea; states within large countries such as Michigan, USA or Victoria, Australia; climatologically-homogeneous regions in Peru; provinces within Thailand; multiple cities in northern Argentina or in Taiwan; and a subtropical island in Japan.

For sites with multiple years of data, the peak influenza month of each year was determined and the average month of peak influenza was calculated. Because some sites were characterized by semi-annual influenza activity, influenza peaks separated by four months or greater were considered distinct influenza seasons (see Supplement for more information). Indeed, 17 sites (22% of the dataset) concentrated in East and South-East Asia, and equatorial regions of Central and South America, were characterized by two distinct influenza peaks within the year (Figure 1). Of these 17 sites with semi-annual influenza peaks, 15 had a primary peak (present in all study years) and a secondary peak (present in a subset of years), while primary and secondary influenza peaks were not distinguishable in two sites. We report results from analyses performed on the superset of sites consisting of all primary and secondary influenza peaks (n = 96), as well as the subset restricted to primary influenza peaks (n = 76).

In addition to the epidemiological influenza database gleaned from the literature and encompassing 78 sites, we collected laboratory-confirmed influenza epidemiological data for 9 countries from FluNet, the WHO global influenza surveillance effort [24], to ensure proper model validation with an independent disease dataset. The group of 9 countries (Spain, Tunisia, Senegal, Philippines, Vietnam, Colombia, Paraguay, South Africa, and Argentina) was selected because they were latitudinally diverse, with a heavy focus on subtropical and tropical regions, each country was relatively small geographically, and provided several years of data. We also favored countries that were not represented in the original 78-location database.

#### Climate data

For the 78 influenza sites, we compiled average monthly temperature (°C), relative humidity (%) and precipitation (mm) data from the CRU/Oxford/IWMI 10′ latitude/longitude gridded dataset (CRU CL 2.0) [25]. This dataset was selected because of its global coverage, high spatial resolution, and monthly temporal resolution (equal to the temporal resolution of the epidemiological dataset). We then calculated the average monthly specific humidity (g/kg)—a measure of absolute humidity—from relative humidity, temperature and surface pressure (estimated from elevation) [14].

Because the CRU climate dataset consists of monthly averages for the arbitrary multiyear period of 1960–1991 and does not necessarily represent the local monthly conditions in the years sampled for influenza activity, we also considered a more recent meteorological dataset from the NCEP/NCAR Global Reanalysis (GR) project [26]. Unlike the CRU dataset, the GR dataset provides comprehensive time series data for 1948-present, which enables calculation of average monthly meteorological conditions for the appropriate time periods at all sites. The major drawback of the GR dataset is its coarser spatial scale (2°×2° latitude/longitude), which can obscure local variability in weather and climate.

To evaluate potential biases associated with a mismatched time period (CRU dataset) and a coarse spatial resolution (GR dataset), we performed a comparative analysis of both datasets (Figure S1 in Text S1). We established that the root-mean-square (RMS) error introduced by the mismatched temporal period was 3–5 times smaller than the RMS error introduced by a coarse spatial resolution. Thus, herein we report the results of analyses based on temperature, specific humidity, relative humidity and precipitation data from the more spatially-resolved CRU dataset. We also use the solar radiation variable from the GR dataset as the CRU dataset does not have appropriate solar radiation information (Supplement). Finally, we obtained climate data from the CRU and GR datasets for the most populous city in each of the 9 countries selected from FluNet for additional model validation.

### Statistical Analyses of Influenza and Climate

#### Exploratory rank analyses

We conducted exploratory analyses based on a non-parametric rank order approach to assess the relative association between influenza peaks and seasonal climate variation. Specifically, we ranked the monthly values of temperature, solar radiation, specific humidity, and precipitation for each site in ascending order. We calculated the mean of the ranks corresponding to the peak influenza month(s) for each site. Assuming that there is no relative relationship between climatic factors and timing of influenza peaks, we would expect mean ranks of 6.5 (“null value”). We also performed the rank order analysis across latitude by employing a window spanning 10° of latitude and sliding it across 5–50°N/S latitude at 2.5° intervals. We calculated the mean rank for each climate variable corresponding to influenza peaks for all the sites within each interval. To test for significance we generated a null distribution (p = 0.05) by bootstrapping randomly generated distributions for each latitudinal interval. We evaluated lag relationships of up to 4 months between climate factors and influenza peaks.

#### Univariate and multivariate regression

To assess the absolute relationship between influenza seasonality and climate, we developed univariate and multivariate logistic regression models. Because exploratory analyses revealed a bimodal relationship between influenza peaks and some of the environmental predictors we employed second-degree polynomial functions for climate predictors. We also added interaction terms describing the deviation of each monthly predictor from its annual average. The dependent variable was a vector of months indicating the presence or absence of an influenza peak for each site and month.

As a sensitivity analysis, we considered mixed effects logistic models with independent intercepts (exchangeable covariance structure) to control for the repeated measurements in each site and different disease periodicities (i.e., annual versus semi-annual influenza activity). We found this did not have a significant effect on the modeled relationships and therefore report the results from classical logistic regression for simplicity.

We used the “all possible subsets” multivariate regression approach and retained models in which all predictors were significant (p = 0.05). In conjunction with a jackknife leave-one-out method we assessed model fit with a “peak prediction metric” that was defined by calculating the difference between the observed month of peak influenza activity versus the month with the highest predicted probability of a peak for each site. The cumulative proportion of peaks predicted within +/−1 month of the observed peaks were compared to the upper 97.5%, 99% and 99.9% thresholds of the cumulative proportion of influenza peaks randomly distributed within +/−1 months of the observed peaks (10,000 runs). In addition to using the peak prediction metric to quantify model performances across all sites, we compared model performances between sites in high latitudes (poleward of 25°N/S), middle latitudes (12.5–25°N/S) and low latitudes (equatorward of 12.5°N/S). We evaluated lag relationships of up to 4 months between the predictors and influenza peaks.

For additional model validation, we confronted the predictions of the selected multivariate climate model against the seasonal distribution of influenza viruses in independent sites selected from the FluNet database.

#### Defining influenza geographical and climatic boundaries

The rank order and logistic regression analyses indicated that the relationship between seasonal climate and influenza peaks was not consistent globally. Low temperatures, solar radiation and specific humidity corresponded to epidemics in high latitudes, whereas high levels of precipitation, specific humidity and relative humidity corresponded to epidemics in low latitudes. To try to synthesize these results, we explored whether the seasonal range of climatic factors in a site was predictive of the environmental conditions during the local influenza season. Specifically, we constructed a binary dependent variable by classifying each influenza peak as either ‘cold-dry’ or ‘humid-rainy’ based on whether the influenza peak corresponded to a month with a specific humidity rank less than or equal to 6 or greater than or equal to 7, respectively. Specific humidity was chosen to classify the peaks because the rank-relationship between influenza peaks in the low latitudes was the opposite of the relationship observed in high latitudes, and because specific humidity was significantly correlated with other relevant climate predictors, including temperature (Pearson rho = 0.87, p<0.0001) and precipitation (Pearson rho = 0.62, p<0.0001). We then used the site-specific annual minimum and maximum of each environmental predictor to generate a conditional probability function through logistic regression. We defined the value at which the function equaled 0.50 to be the threshold between cold-dry and humid-rainy influenza locations. We used a jackknife leave-one-out method to assess the accuracy of the logistic model in predicting the climate conditions corresponding to each influenza peak. Finally, for additional model validation, we confronted the predicted probability function against observed influenza activity patterns in the independent study sites selected from FluNet.

## Results

### Influenza Peaks and Climate: Rank Analysis

Across the 78 sites, influenza peaks generally coincided with months of lower temperature, lower solar radiation and lower specific humidity than expected under the null hypothesis (mean rank = 4.3 [95% CI: 3.7, 5.0] for temperature, 4.5 [95% CI: 3.9, 5.1] for solar radiation and 4.8 [95% CI: 4.0, 5.6] for specific humidity). In contrast, relative humidity and precipitation did not significantly deviate from the null value (mean rank not significantly different than 6.5). The association between influenza peaks, temperature, solar radiation, relative humidity and specific humidity was most significant when the influenza peaks lagged 1-month behind the environmental predictors. We obtained similar results when the analysis was restricted to primary influenza peaks.

**Figure 2:**
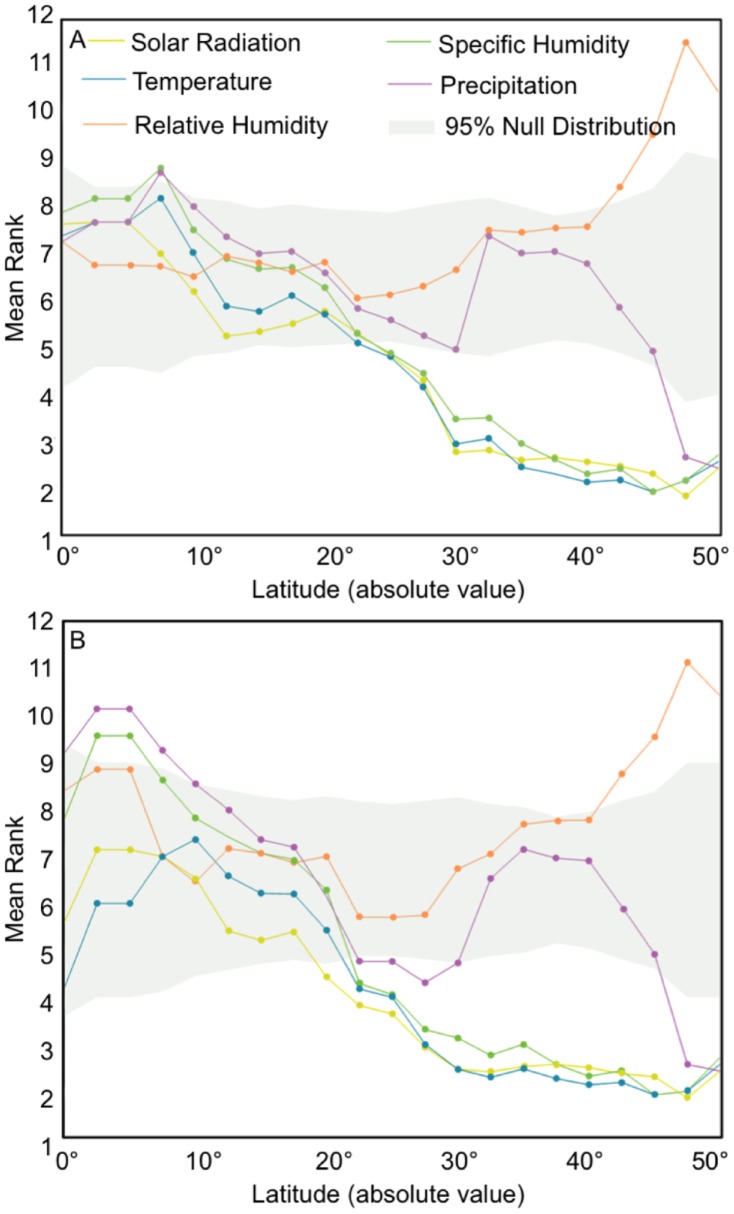
Influenza peaks and climate by latitude. The mean monthly rank of each climate variable corresponding to the month of peak influenza for each 10° latitudinal band. Solar radiation, temperature and specific humidity are lagged by 1 month. The background interval corresponds to the 95% null distribution. (A) displays the results for both primary and secondary influenza peaks; whereas (B) shows the results for primary influenza peaks only. Influenza peaks corresponded to months characterized by low ranks of temperature, solar radiation, and specific humidity in high latitudes. Primary influenza peaks corresponded to months with high ranks of humidity (both relative and specific) and precipitation in low latitudes.

A similar analysis performed with a sliding geographical window revealed that the association between influenza peaks and climatic variables varied with latitude (Figure 2). The strongest association was found at high latitudes poleward of 25°N/S, with influenza peaks preferentially occurring in months with the lowest temperature, solar radiation and specific humidity. Influenza peaks occurred in the months with the highest levels of relative humidity and lowest levels of precipitation poleward of approximately 40°N/S. Primary influenza peaks equatorward of 10°N/S corresponded to the months with the highest annual levels of specific humidity and precipitation (p<0.05); whereas there was no association with temperature, solar radiation and relative humidity. In middle latitudes ranging between 12.5–25°N/S, there was no significant association between influenza peaks and climatic variables.

### Univariate and Multivariate Models for Influenza Peaks

**Figure 3:**
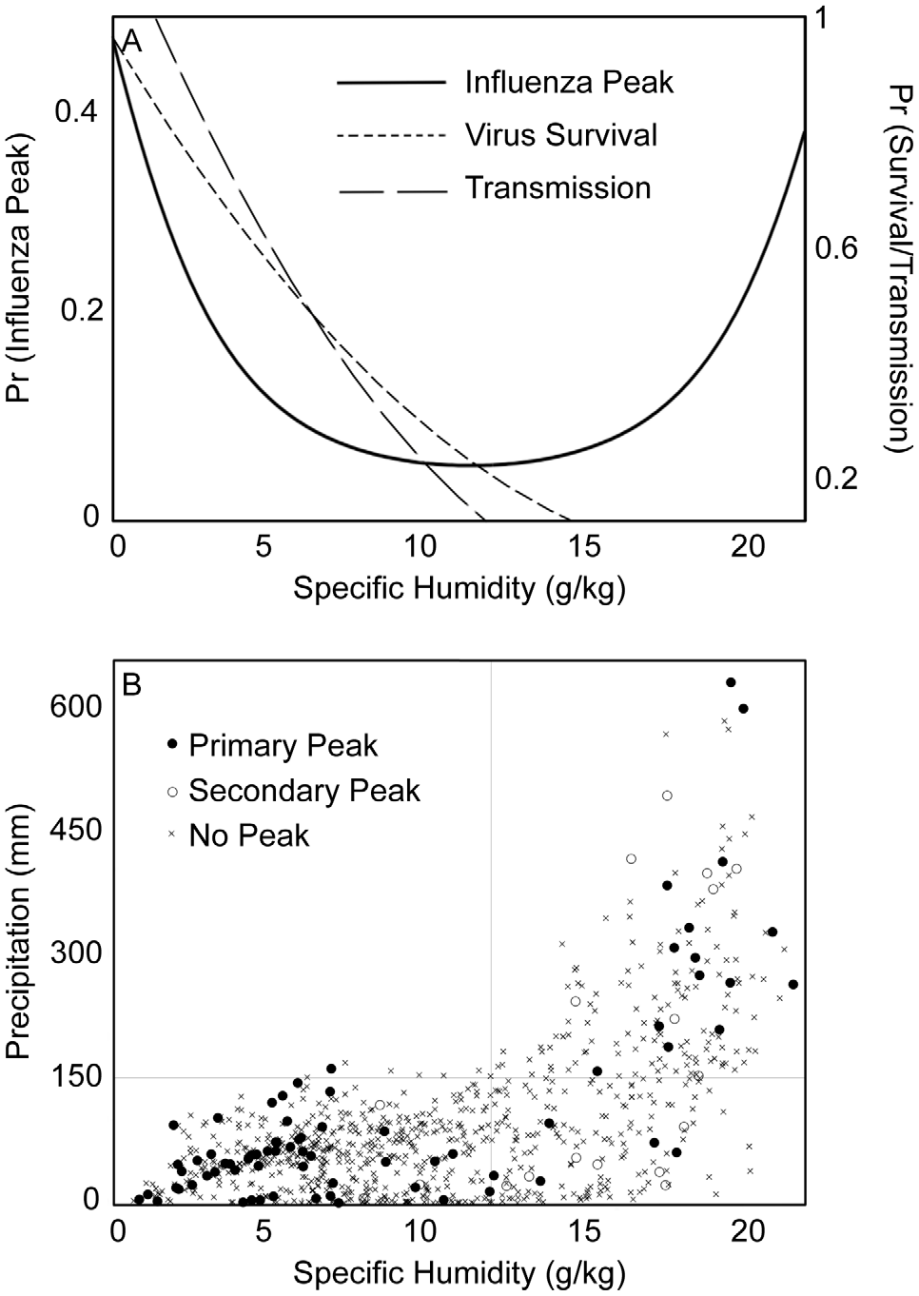
Influenza peaks, specific humidity and precipitation. (A) Estimated U-shaped relationship between the likelihood of an influenza peak and average monthly specific humidity across all sites, based on logistic regression (Table 1). The left side of the curve is strongly correlated with the relationship between specific humidity and influenza survival and transmission observed in laboratory studies [17], [32], [33]. However, the mechanism that causes the pattern on the right side of the curve is not readily explained. (B) The relationship between average monthly specific humidity and precipitation across all sites. Influenza peaks clustered in months associated with low specific humidity and high precipitation conditions. This suggests that precipitation may explain the occurrence of humid-rainy influenza peaks and may be responsible for the right hand side of the U-shaped curve between specific humidity and influenza (A).

Temperature and specific humidity were the best individual predictors of influenza peaks. The model fits improved slightly when influenza peaks lagged 1-month behind these predictors, and accurately predicted 56–66% of the peaks in the global datasets, with highest accuracy at latitudes poleward of 25°N/S (Tables 1 and 2). The modeled relationship between specific humidity and all influenza peaks was U-shaped, with lowest probability of an influenza peak at 12 g/kg of specific humidity and increasing probabilities at lower and higher values (Figure 3). The analysis restricted to primary peaks revealed a similar relationship, with a minimum influenza probability at 11 g/kg. Unlike specific humidity the modeled relationship between temperature and influenza peaks was monotonic, with the greatest probability of a peak corresponding to low temperatures. Although the specific humidity and temperature models were the best predictors of the timing of influenza peaks across all sites, they were not significant predictors of influenza peaks equatorward of 25°N/S.

**Table 1:** Environmental models for all influenza peaks, by latitudinal interval.

			Proportion of peaks accurately predicted by each model			
Climatologies	Coefficients (SE)	AIC	All Peaks n = 96	aHigh Latit. n = 50	bMiddle Latit. n = 31	cLow Latit. n = 15
**Temperature (Precipitation)^2^**	−0.05 (4.66e-6) 1.10e-5 (7.78e-5)	590	0.61***	0.82***	0.39	0.40
**Temperature**	−0.03 (0.01)	611	0.59***	0.84***	0.39	0.20
**Specific Humidity (Specific Humidity)^2^**	−0.54 (−0.09) 0.02 (3.82e-3)	593	0.56***	0.78***	0.32	0.33
**(Solar Radiation)^2^**	−2.06e-5 (4.18e-6)	578	0.52***	0.70***	0.39	0.20
**Relative Humidity**	0.03 (0.01)	613	0.41*	0.52***	0.19	0.47
**(Precipitation)^2^**	7.28e-06 (1.95e-6)	615	0.31	0.22	0.39	0.47
**Expected Values i.e. null distribution (95% CI)**			0.25 (0.16 0.34)	0.25 (0.14 0.38)	0.24 (0.09 0.42)	0.25 (0.00 0.53)

* p<0.05,

** p<0.01,

*** p<0.001.

a high latitudes are regions poleward of 25°N/S.

b middle latitudes are regions between 12.5°N/S and 25°N/S.

c low latitudes are regions equatorward of 12.5°N/S.

The results of selected logistic regression models, based on Aikake Information Criterion (AIC) and the proportion of peaks accurately predicted by each model using a jackknife leave-one-out method. These values can be compared against the expected values and corresponding confidence intervals under the null distribution in the bottom row. The models are in descending order based on the proportion of peaks accurately predicted. Influenza peaks were lagged by 1-month with respect to each environmental variable with the exception of precipitation.

**Table 2:** Environmental models for primary influenza peaks.

			Proportion of peaks accurately predicted by each model			
Climatologies	Coefficients	AIC	All Peaks n = 76	aHigh Latit. n = 47	bMiddle Latit. n = 20	cLow Latit. n = 9
**Temperature Specific Humidity**	−0.10 (2.56e-3) 0.10 (1.76e-3)	501	0.70***	0.87***	0.30	0.67**
**Temperature**	−0.05 (0.01)	504	0.66***	0.85***	0.35	0.33
**Specific Humidity (Specific Humidity)^2^**	−0.55 (0.09) 0.02 (4.18e-3)	493	0.62***	0.81***	0.35	0.22
**(Solar Radiation)^2^**	−3.50e-5 (5.14e-6)	471	0.59***	0.72***	0.40	0.33
**Relative Humidity**	0.02 (0.01)	521	0.43***	0.53***	0.10	0.67**
**(Precipitation)^2^**	5.24e-06 (2.57e-6)	524	0.32	0.21	0.40	0.67**
**Expected Values i.e. null distribution (95% CI)**			0.24 (0.13 0.38)	0.24 (0.16 0.36)	0.20 (0.05 0.45)	0.20 (0.00 0.55)

* p<0.05,

** p<0.01,

*** p<0.001.

a high latitudes are regions poleward of 25°N/S.

b middle latitudes are regions between 12.5°N/S and 25°N/S.

c low latitudes are regions equatorward of 12.5°N/S.

Same as Table 1 but these are the results for primary influenza peaks only.

There was a strong inverse relationship between solar radiation and the probability of an influenza peak, especially when influenza peaks were lagged by 1-month. The solar radiation model outperformed the temperature and specific humidity models based on AIC, but it was not as strong a predictor of the timing of the influenza peaks (Tables 1 and 2).

Precipitation was a weak predictor of influenza peaks overall, but it was a strong predictor of influenza peaks equatorward of 12.5°N/S, particularly for primary influenza peaks (p<0.01) (Tables 1 and 2). Unlike the other climate variables, precipitation-based models performed slightly better when no lag was considered between precipitation and influenza activity.

Relative humidity was a strong predictor of influenza peaks globally, particularly when a 1-month lag was applied to the influenza peaks. There was a positive association between relative humidity and influenza peaks in high and low latitudes, but this model was a poor predictor of influenza peaks in middle latitudes. Further, the relative humidity model was not as strong as the specific humidity, solar radiation and temperature models (Tables 1 and 2).

**Figure 4:**
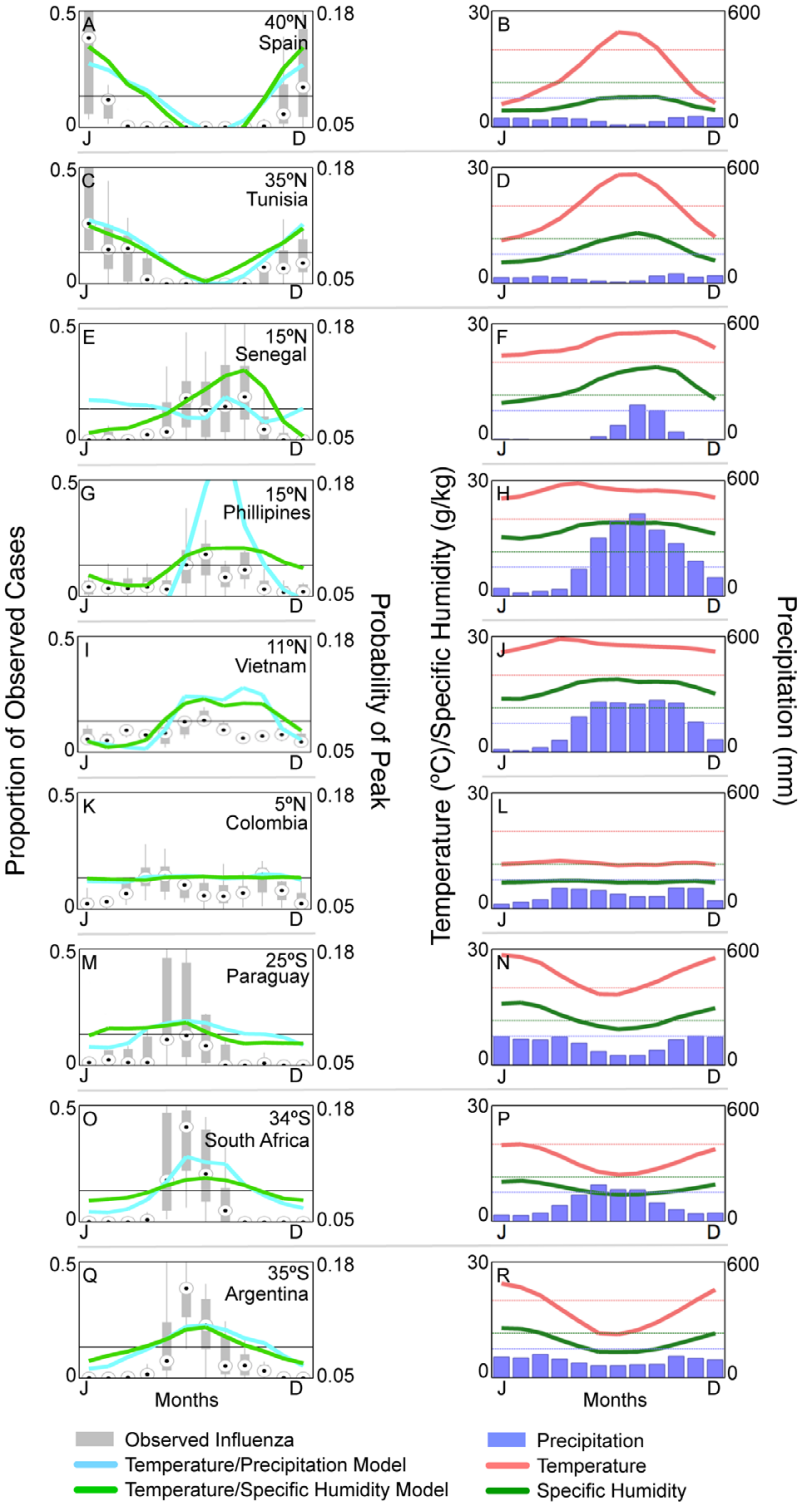
Influenza seasonal distribution for 9 sites selected from an independent epidemiological dataset and climate model outputs. (A,C,E,G,I,K,M,O,Q) Box plots indicate the proportion of influenza cases occurring in each month of the year for 9 countries with multiyear data selected from FluNet. Results of the best-fit climate models for all and primary peaks (Tables 1 and 2) are displayed for comparison. Specific humidity and temperature were advanced one month to account for the one month lag between influenza peaks and these variables. Although the models were designed to estimate the timing of peak influenza activity, they also provide estimates of the seasonal distribution of influenza virus circulation. (B,D,F,H,J,L,N,P,R) The right column displays the monthly precipitation, temperature and specific humidity for each location. Dotted lines indicate the climatic thresholds for each variable. In general, when temperature or specific humidity drops below their respective thresholds, or precipitation surpasses its threshold, there is an increase in influenza activity.

Overall, the multivariate models most predictive of influenza peak timing included combinations of temperature and precipitation (all peaks, Table 1), and temperature and specific humidity (primary peaks, Table 2). These models accurately predicted peak influenza months in 78% and 89% of the 9 independent sites selected from the FluNet database, respectively (Figure 4). Further, the models predicted a nearly uniform probability of influenza peaks every month of the year in equatorial Colombia, a location that experiences minimal seasonal climatic fluctuation (Figure 4). Taken together, this analysis exploring the shape of the relationship between climatic variables and influenza highlights the covariability between specific humidity and temperature, and the significant predictive power of these variables at high latitudes. In contrast, precipitation and relative humidity were predictive of influenza peaks at low latitudes. Interaction terms describing monthly deviations from the annual average of each environmental predictor marginally improved some models but did not affect the main conclusions (Tables S1 and S2 in Text S1).

### Geographic and Climatic Boundaries Predictive of Influenza Peaks

**Figure 5:**
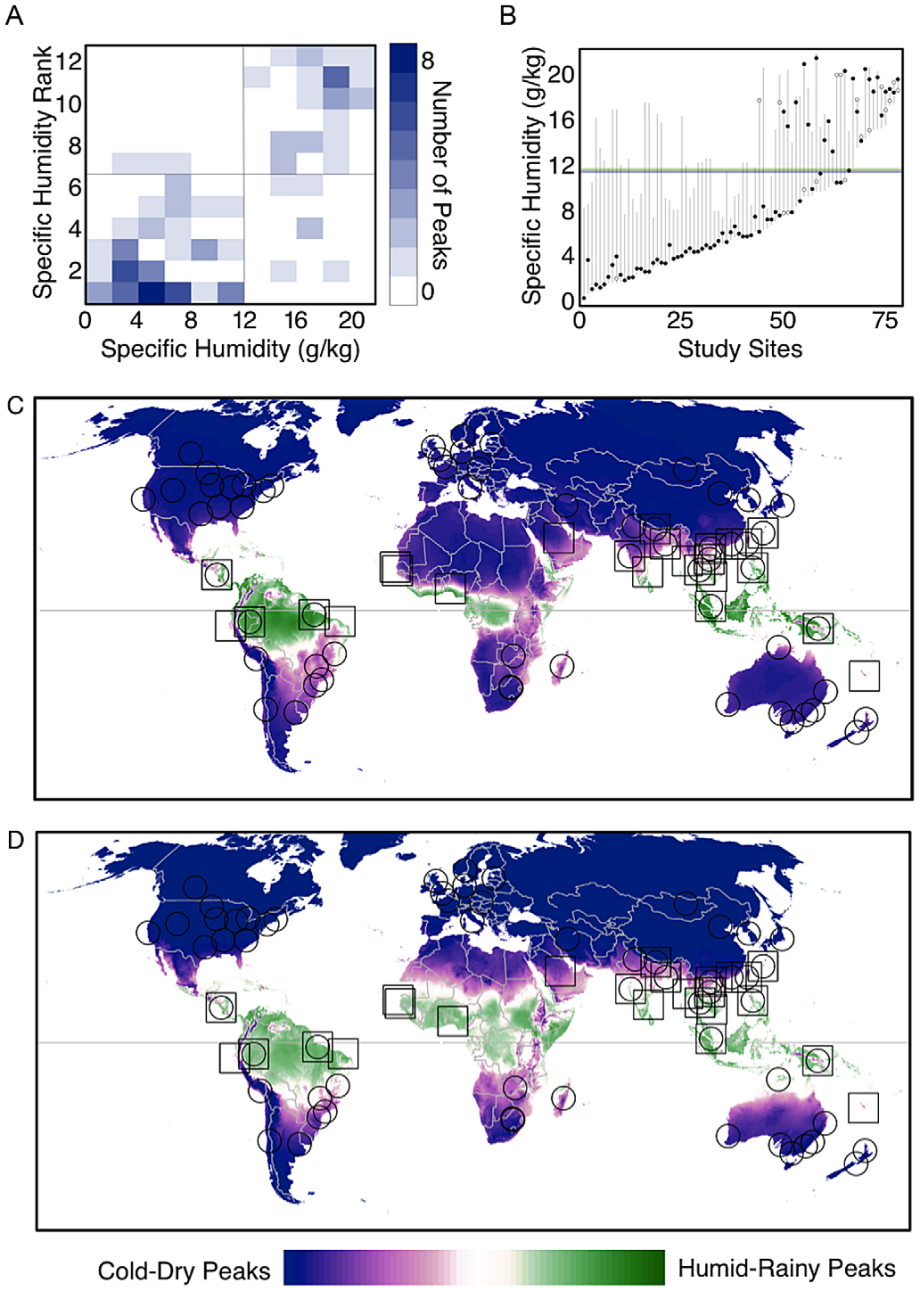
Climatic thresholds predictive of influenza seasonal characteristics. (A) density plot showing the specific humidity in absolute terms (x-axis) and relative terms (y-axis) during influenza peaks across all sites. The plot shows that a vast majority of influenza peaks occurred in “cold-dry” conditions when specific humidity was lower than 8 g/kg and ranks were less than 4, or during “humid-rainy” conditions when specific humidity was greater than 14 g/kg and ranks were greater than 9. (B) a line plot showing the average annual range of specific humidity (y-axis) for each location (x-axis). Sites are ordered based on minimum specific humidity. The black dots indicate the specific humidity during the month of the primary peak and circles indicate specific humidity during secondary peaks. Together, the plots suggest that sites with the lowest annual minimum specific humidity have influenza peaks when specific humidity is at locally-minimal levels. (C) a map displaying the predictions of a logistic regression indicating the probability of an influenza peak during the cold-dry season, versus the humid-rainy season, based on annual minimum specific humidity. The markers indicate the 78 study sites with influenza peaks classified as cold-dry (circles) and humid-rainy (squares). (D) same as (C) but the model is based on annual minimum temperature.

To further characterize the distribution of influenza peaks globally and identify the geographical and climatic boundaries defining influenza seasonality patterns, we categorized sites based on whether influenza epidemics occurred in months with low (cold-dry season) or high (humid-rainy season) levels of specific humidity relative to the local climatology (Figure 5). We found that the annual minimum level of specific humidity in a site was predictive of the seasonal characteristics of influenza activity locally. Sites characterized by cold-dry influenza peaks generally experienced annual minimum specific humidity values less than 12 g/kg when all influenza peaks were considered, and approximately 11 g/kg when the analysis was restricted to primary influenza peaks (Figure 5). The minimum specific humidity models were statistically significant, and classified 75% of the 96 total influenza peaks correctly (p<0.001, Figure 5C), and 82% of the 76 primary peaks correctly (p<0.001). Annual minimum temperature was a slightly better predictor of the type of influenza peak characterizing a site, correctly classifying 77% and 87% of all peaks and primary peaks, respectively (p<0.001). Sites characterized by cold-dry influenza peaks generally had annual minimum temperature values less than 21°C when all influenza peaks were considered, and approximately 18°C when the analysis was restricted to primary influenza peaks. The temperature and specific humidity models differentiated between the 6 cold-dry and 3 humid-rainy influenza peaks available in the independent FluNet sites with 100% and 78% accuracy, respectively.

Annual minimum solar radiation was a significant predictor of the type of influenza peaks, comparable to temperature and specific humidity, correctly classifying 71% and 79%of all peaks and primary peaks, respectively (p<0.001). Annual minimum relative humidity and annual monthly maximum precipitation were also significant predictors, but the models were significantly weaker than the other models. It should be noted that 12 sites had both a cold-dry and humid-rainy influenza peak, assuring that one peak would be classified incorrectly when all influenza peaks were considered.

Taken together, this analysis indicates that thresholds in specific humidity and temperature, and perhaps solar radiation, are associated with the timing of the influenza season and the occurrence of influenza activity in the dry-cold or humid-rainy months of the year. The specific humidity and temperature models were then used to predict the expected seasonal characteristics of influenza globally (Figure 5C–D). Both models suggest that seasonal influenza activity coincides with the humid-rainy season in large areas of Central and South America, and Southern Asia; while predictions were more uncertain in middle latitudes and there were inconsistencies between the two models for parts of Central Africa. In particular, the model driven by minimum temperature predicted the occurrence of humid-rainy influenza peaks in most of Central Africa, while the model driven by minimum specific humidity predicted a more restricted zone of humid-rainy peaks concentrated on the Western coast of this region. These discrepancies can be explained by a combination of warm year-round temperatures in this area, with low specific humidity values in parts of the year.

## Discussion

We explored the association between influenza seasonality and climate in a representative sample of 78 global sites, spanning an absolute latitudinal range between 1° and 60°. Our analyses revealed two distinct types of climatic conditions associated with influenza seasons observed globally: “cold-dry” and “humid-rainy”. In general, sites that experienced low levels of specific humidity and temperature (less than 11–12 g/kg and 18–21°C) for at least one month during the year were characterized by seasonal influenza activity during the months with minimal levels of specific humidity and temperature. In contrast, sites that maintained high levels of specific humidity and temperature were generally characterized by influenza epidemics during the most humid and rainy months of the year. The predictions of our climate-based models compared favorably to influenza epidemiological information collected independently of the dataset used for the model-building exercise.

The bimodal nature of the relationship, in both relative and absolute terms, between specific humidity and influenza peaks, and its strong relationship to other climate variables such as temperature and precipitation, makes specific humidity a useful gauge of the environmental favorability of influenza activity across all latitudes (Figure 5). However, although the specific humidity models were significant predictors of influenza peaks globally, this was primarily due to their performance in high latitudes. In low latitudes, precipitation was a stronger predictor of the timing of influenza activity, with peaks typically occurring in months with average precipitation greater than 150 mm (Figure 3).

Overall, although precipitation was strongly associated with influenza peaks in low latitudes, the timing of influenza peaks in this region was more difficult to predict than in high-latitude sites. Several sites in this region were not characterized by well-defined influenza season; rather, influenza activity was present year-round likely due to the limited seasonal environmental variation that characterizes much of the region. For example, equatorial sites such as Iquitos, Peru, and Singapore—where influenza seasonality is weak [27], [28]— experience limited fluctuations in precipitation, with monthly averages constrained to a narrow range of 150–300 mm year-round. In contrast, middle and low-latitude sites such as Fortaleza, Brazil and Yangon, Myanmar —which are noted for their well-defined influenza seasons [9], [11]—are characterized by large amplitude range in average monthly precipitation from 25 mm in the dry season to over 300 mm and 600 mm in the rainy season, respectively.

Model performances were particularly poor in a number of middle latitudes sites. Predicting influenza peaks in these sites may be complicated by large seasonal swings in climate that characterize the region, generating both cold-dry and humid-rainy seasons that are equally favorable for seasonal influenza activity, such as in Senegal (Figure 4). For these sites other factors might play a critical role in determining the timing of influenza activity, including population mixing (i.e., travel) with regions that do experience well-defined influenza seasons [29], [30], or the seasonal phasing with school cycles [21]. Moreover, the presence of both cold-dry and humid-rainy seasons could explain the occurrence of semi-annual influenza epidemics in some of these middle-latitude sites. For example, Hong Kong has a primary influenza peak in winter when average monthly specific humidity and temperature are less than 8 g/kg and 17°C, and a secondary influenza peak in summer when average monthly precipitation is near 400 mm.

Temperature was a strong predictor of influenza seasonality in high latitudes, suggesting that cold temperatures may drive seasonal epidemics in these regions. However, previous analyses of laboratory experiments have indicated that specific humidity is a more parsimonious predictor of virus survival and transmission than temperature [17]. Furthermore, individuals in temperate regions spend a vast majority of their time indoors where temperature is managed and does not correlate well with outdoor temperatures. Yet, temperature may affect the timing of influenza epidemics through mechanisms independent of virus survival; for example, low outdoor temperatures may promote indoor crowding, thereby increasing person-to-person contact rates [2]–[4]. It is also possible that even limited exposure to cold outdoor temperatures can have long-lasting physiological effects on hosts that make them more susceptible to infection or affect viral shedding [16]. Additional experimental and observational work is needed to disentangle the contribution of specific humidity and temperature on influenza seasonality; epidemiological information from Central Africa would be particularly useful in this respect as our climate-based predictive models disagreed in this region.

The findings that both cold-dry and humid-rainy conditions are associated with influenza peaks could be used to support the hypothesis that two distinct mechanisms account for influenza seasonality in temperate and tropical climates, perhaps due to changes in the dominant mode of transmission [31]. For example, specific humidity may drive the timing of influenza epidemics in high latitudes by increasing virus survival and enabling aerosol transmission; whereas direct transmission or transmission by fomites may dominate in low-latitude sites where rainy conditions favor indoor crowding. Middle latitudes may be a transition zone where influenza seasons are driven by low specific humidity or high levels of precipitation depending on local climate. Another intriguing possibility is that the relationship between specific humidity and virus survival underlies influenza transmission across all latitudes. For example, a few experimental studies have indicated a U-shaped relationship between relative humidity and influenza virus survival, suggesting a similar relationship for specific humidity given that experiments were held at constant temperature [32]–[34]. Other laboratory studies, however, have indicated that virus survival and transmission increase monotonically as specific humidity decreases [17], [35], [36]. Further, the hypothesis that specific humidity drives influenza transmission globally is inconsistent with the low predictive power of this climatic variable in middle and low-latitude sites in our study.

Relative humidity was a strong predictor of influenza peaks in high and low latitudes, but a poor predictor in middle-latitude. In high-latitude regions, relative humidity can vary significantly between indoor and outdoor environments, and it is typically minimal indoors during the winter when building air is heated. Our analysis relied on outdoor humidity and hence we cannot rule out that winter influenza epidemics in high latitudes could be related to low indoor relative humidity and associated changes to host physiology, such as reduced mucociliary clearance [20]. In low latitudes it is possible that relative humidity is confounding precipitation in our analysis. Disentangling these two factors will require more highly-resolved epidemiological data from equatorial regions, and further experimental and observational studies.

Solar radiation was also a significant predictor of influenza peaks in high latitudes suggesting that it may also underlie influenza seasonality in these regions, perhaps through variation in vitamin D intake [18]. However, solar radiation was not as strong a predictor of influenza peaks as were specific humidity and temperature. This corroborates recent studies indicating that specific humidity is a stronger predictor of seasonal influenza activity than solar radiation and vitamin D variability in the U.S. [14], [37]. Still, the potential seasonal forcing of solar radiation on influenza transmission warrants further experimental and observational investigation.

The power of this study was rooted in the large number of spatially diverse sites used to develop the epidemiological and climatic databases and associated models. However, the challenge of describing seasonal influenza activity consistently across a variety of data sources required a crude epidemiological measure, such as the average month of peak influenza activity. This measure of influenza activity has several key drawbacks. Foremost, all months with the exception of the peak influenza months were considered equal, whether they had substantial influenza activity or not. Second, the month of peak influenza activity may not be contemporaneous with the month in which transmission is under the most environmentally favorable conditions, since non-environmental factors such as viral seeding, population susceptibility, and person-to-person contact rates likely play a role in the timing of influenza epidemics [15], [21]. In this respect, it is reassuring that a 1-month lag maximized the association between influenza peak and most of the climatic variables, which is broadly consistent with the time scale of the ascending phase of a local epidemic. Third, we could not assess putative geographical variation in the transmission potential or intensity of influenza epidemics. For example, we may expect locations that have the most favorable environmental conditions to experience the greatest influenza annual attack rates and reproduction numbers, holding all other relevant variables equal. A further limitation relates to between-year variability in influenza timing and the limited temporal sampling of our dataset, which may have resulted in imprecise estimates of the average influenza peak in some sites, especially sites that had only one year of influenza data. However, sensitivity analyses limited to multi-year studies revealed similar relationships between climate predictors and influenza peaks, confirming the robustness of our results. Finally, we were unable to check whether between-year fluctuations in climatic variables may result in departures from average influenza seasonal characteristics in specific years. This question could be an interesting area for future research with more temporally refined epidemiological datasets.

A number of follow-up studies could help refine our understanding of the small and large-scale processes underlying influenza seasonality. For example, experimental infections in humans under controlled temperature and humidity conditions could determine which environmentally-mediated mechanisms are most important for human-to-human transmission. However, there are several ethical and methodological hurdles to overcome in such studies [38]. Seasonal fluctuation in contact rates could be monitored by wireless sensor technology, which has recently proved successful in estimating dynamic contact patterns in schools and at conferences [39]–[40]. On a broader spatial scale, determining regional differences in influenza transmission dynamics and attack rates would be most informative. A recent study has suggested that the reproduction number of seasonal epidemics was lower on average in Brazil than in temperate countries, which could be mediated by environmental factors [41]. Modeling of long-term influenza time series data could help assess the transmission impact of seasonal fluctuations in population mixing in different regions, such as those associated with school cycles [21] and transportation networks [29], [30], [42]. For example, epidemiological evidence indicates that influenza circulation was weakly seasonal in Iceland prior to the 1930s, presumably because of low connectivity with other populations, and epidemics only became fully synchronized with those in Europe and the USA following a dramatic increase in international travel in the 1990s [43]. Hence, efforts to collate multiyear influenza epidemiological information retrospectively and prospectively in various regions of the globe, especially from middle and low latitude regions, will be of tremendous help to further elucidate the environmental and population drivers of seasonality.

In conclusion, our study broadens our understanding of the relationships between seasonal influenza epidemics and environmental factors and provides a synthesis of epidemiological and climatic characteristics across temperate, subtropical and tropical regions. We have highlighted the importance of thresholds in specific humidity, temperature and precipitation that are associated with the epidemiology (and potentially the modes of transmission) of influenza. The results of this study could help improve existing influenza transmission models by providing a more accurate estimate of the environmental forcing on transmission processes, particularly in low and middle latitudes [14], [44]. Further, our models could be used to predict the seasonal timing of influenza activity in locations with little or no observational data on influenza activity, and help target surveillance efforts and optimize the timing of seasonal vaccine delivery, [45]. More broadly, we hope that our work will generate interest in testing the association between climatic patterns and infectious disease across a wide range of diseases and latitudes, particularly for respiratory and enteric pathogens that display marked seasonality [2], [46]. A better understanding of the environmental, demographic and social drivers of infectious disease seasonality is key for improving transmission models and optimizing interventions [47].

## Supporting Information

Text S1 **Additional information regarding data and results.** Included is a more detailed description of the influenza database and the algorithm developed to define each “peak”. We also provide a comparison of CRU and GR climate datasets, and provide results from additional predictive models of peak influenza timing.
(DOC)
