# Correction: Environmental Predictors of Seasonal Influenza Epidemics across Temperate and Tropical Climates

**DOI:** 10.1371/annotation/df689228-603f-4a40-bfbf-a38b13f88147

**Published:** 2013-11-21

**Authors:** James D. Tamerius, Jeffrey Shaman, Wladimir J. Alonso, Kimberly Bloom-Feshbach, Christopher K. Uejio, Andrew Comrie, Cécile Viboud

In the author list, the spelling of the name Wladmir J. Alonso is incorrect.

The correct spelling is Wladimir J. Alonso. 

