# Supplementary material for: Environmental Predictors of Seasonal Influenza Epidemics across Temperate and Tropical Climates
Source: PLoS Pathog. 2013 Mar 7;9(3):e1003194. doi: 10.1371/journal.ppat.1003194 (PMC3591336; doi:10.1371/journal.ppat.1003194)
Supplement: Text S1 — Additional information regarding data and results. Included is a more detailed description of the influenza database and the algorithm developed to define each “peak”. We also provide a comparison of CRU and GR climate datasets, and provide results from additional predictive models of peak influenza timing. (DOC) [file ppat.1003194.s001.doc]

**Environmental Predictors of Seasonal Influenza Epidemics**

**Across Temperate and Tropical Climates: Supplementary Material**

James D Tamerius^1,2^* Jeffrey Shaman^1,2^ Wladmir J Alonso^2^ Kimberly Bloom-Feshbach^3^ Christopher K Uejio^4^ Andrew Comrie^5^ Cécile Viboud^2^

**1. Supplementary Data: Detailed description of influenza database**

To generate the influenza database a literature search was conducted in PubMed between May 2009 and February 2010 for influenza articles published between 1990 and 2009. The keyword “influenza” was used in combination with the epidemiological terms “laboratory” and “surveillance”, and geographical terms such as “tropics”, “temperate”, “Asia”, “Europe”, “Middle East”. The literature review was restricted to papers in English. References cited in retrieved articles were also examined for possible inclusion in this review. We included all hospital and community-based studies that reported at least 24 laboratory-confirmed influenza cases per year (i.e, more than 2 each month), were conducted for one consecutive year or more, and reported weekly or monthly counts or percent of virus positive in a table or graphic format. We excluded studies from the 2009 A/H1N1 pandemic to limit the analysis to influenza patterns in inter-pandemic periods. In addition to published literature, we also obtained publicly available influenza virological data from international and national influenza surveillance systems, including the Australia Department of Health’s National Influenza Surveillance Scheme[1], the European Influenza Surveillance Scheme [2], Japan’s Infectious Disease Surveillance Center [3], the World Health Organization FluNet [4] and the US influenza surveillance system maintained by the Centers for Disease Control and Prevention [5].

**2. Supplementary Methods: Definition of peak influenza timing**

The seasonal parameter of interest was the month where influenza activity was most intense. Specifically, the peak month was defined as the month with the maximum number of viruses isolated within a calendar year. We took the average peak month when the study covered multiple years. For studies reporting weekly data, the month corresponding to the week of peak activity was recorded.

Some locations were characterized by two distinct periods of influenza activity within the year. We identified locations experiencing semi-annual activity based on whether two peaks in viral isolates occurred at least 4 months apart within the same calendar year. To eliminate the possibility that locations characterized by long influenza seasons (i.e., greater than 4 months) were not erroneously defined as having semi-annual peaks, we required that the number of laboratory confirmed cases had to decrease below 5% of the annual total between the two distinct peaks. In all locations with semi-annual activity, we could define a primary peak (highly consistent between years) and a minor peak (less intense and/or less consistent), except for Singapore.

**3. Sensitivity analyses: Comparison of CRU and GR Climatic Datasets**

This analysis compares the two climatic databases that were available for study: the Climate Research Unit CL 2.0 (CRU) and NCEP Global Reanalysis datasets (GR).

Because of its higher spatial resolution, we used the CRU dataset in the main analysis for relative humidity, specific humidity, precipitation, and temperature, whereas we used the solar radiation from the GR dataset. Solar radiation from the GR was used because the CRU only contains a variable that describes the percent of maximum sunshine possible given the day length. Given that we were mainly interested in the exposure of individuals to solar radiation, which is dependent on day length and solar intensity, we did not believe the CRU variable was suitable for this analysis. Instead, we used the GR data which provides downward solar radiation flux in W/m^2.

Because the CRU climate dataset consists of monthly averages for an arbitrary multiyear period (1961-1990) it does not necessarily provide the best representation of the average monthly conditions for the epidemiological data, which covers the period 1975- 2008. On the other hand, the GR dataset has a coarse spatial resolution (2º x 2º latitude/longitude) but allows the selection of the months that corresponded to the time period of the epidemiological data from each site. Below we assess the biases that are introduced into the data due to the spatial and temporal characteristics of the CRU and GR dataset.

We generated three distinct subsets of the GR and CRU data that describe the average climate conditions for each site and month of the year. The first subset (GR_mtchd_) was generated by extracting data from the GR that matched the time periods of the studies used to estimate the influenza peaks. It should be noted that 2 locations had to be dropped from this subset because the period the epidemiological data were collected was not reported (all from [6]). For comparison, we constructed another GR subset (GR_cl_) that was based on the same 30 year period as the CRU data (1961-1990). The third subset used is the climatic data that was described in the manuscript, i.e., a combination of GR (for solar radiation) and CRU (for the other climatic variables).

The monthly values in the GR_mtchd_ and GR_cl_ were highly correlated (Figure S1) for temperature (R = 0.995, p <0.0001), and relative humidity (R = 0.988, p <0.0001), specific humidity (R = 0.996, p <0.0001) and precipitation (R = 0.971, p <0.0001). The root mean square error (RMSE) across the GR_mtchd_ and GR_cl_ for each climate variable were 0.7 ºC, 2.7 % and 0.4 g/kg and 23 mm, respectively. This indicates that using an arbitrary 30-year period to define the seasonal signal of climate in a location instead of the shorter observational period introduces some error, but that this temporal error is nominal relative to geographical variation in climatic variables across the 73 locations studied.

To explore the effect of spatial resolution of each climatic dataset, we compared monthly values across the GR_cl_ and CRU. Although all the variables were significantly correlated (p < 0.0001), the discrepancies between the monthly values across datasets were much larger than those between the GR_mtchd_ and GR_cl_. Specifically, the RMSE across the subsets are 3.2 ºC, 12.7 %, 2.3 g/kg and 69 mm for temperature, relative humidity, specific humidity and precipitation, respectively. These deviations are 3-5 times larger than those between the GR_mtchd_ and GR_cl_ (Figure S2). In other words, there is more uncertainty introduced by the coarser spatial resolution of the GR dataset, than by the mismatched time period of the CRU dataset.

The result of this sensitivity analysis is not surprising. In general, the seasonal signal of climate is highly consistent across years. Although weather can be variable from year-to-year, when conditions are averaged across time periods as large as a month they tend to converge towards the long term mean; whereas climatological gradients across space can be steep, particularly in mountainous and coastal regions. When the scale of aggregation across such areas is increased information about this variability is lost, which is the case with GR. Overall, this analysis confirms that the error due to the mismatched temporal periods was substantially smaller than the error introduced by the coarser spatial resolution of the GR dataset. This justifies use of the CRU data in this analysis whenever possible.

REFERENCES

1. <http://www.health.gov.au/internet/wcms/publishing.nsf/Content/cda-pubs-annlrpt->fluannrep.htm (Accessed July 1, 2012) Australian Government Department of Health and Aging, Influenza - National Influenza Surveillance Scheme Annual reports.

2. <http://www.eiss.org/index.cgi> (Accessed July 1, 2012) European Influenza Surveillance Scheme (EISS).

3. <http://idsc.nih.go.jp/index.html> (Accessed July 1, 2012) Japan‘s Infectious Disease Surveillance Center.

4. <http://www.who.int/flunet> (Accessed Oct 12, 2011) World Health Organization FluNet.

5. <http://www.cdc.gov/flu/weekly/fluactivity.htm> (Accessed July 1, 2012) CDC National Respiratory and Enteric Virus Surveillance System.

1. Hampson, A. W. (1999). Epidemiological data on influenza in Asian countries. (Vol. 17, pp. S19–23). Presented at the Vaccine.

**Table S1: Additional environmental models for all influenza peaks (n=96), considering monthly deviation from annual means as predictors rather than monthly climatic values. Results of selected logistic regression models with interaction terms as shown, based on AIC and the proportion of peaks accurately predicted by each model using a jackknife leave-one-out method. These values can be compared against the expected values and corresponding confidence intervals (i.e., the null distribution) in the bottom row. The models are in descending order based on the proportion of peaks accurately predicted. Influenza peaks were lagged by 1-month with respect to each environmental variable with the exception of precipitation.**

|  |  |  | **Proportion of peaks accurately predicted by each model** | | | |
| --- | --- | --- | --- | --- | --- | --- |
| **Climatologies** | **Coefficients (SE)** | **AIC** | **All Peaks**  **n = 96** | **High Latit.**  **n = 50** | **Middle Latit.**  **n = 31** | **Low Latit.**  **n = 15** |
| **SH^2^**  **(SH^2^ - SH^2^mean)**  **SH^2^ * (SH^2 -^ SH^2^mean)** | 0.03e-1 (0.01e-1)  0.55 (0.07)  -0.01e-1 (0.03e-2) | 558 | 0.56*** | 0.80*** | 0.35 | 0.20 |
| **T**  **(T - Tmean)**  **T* (T -Tmean)** | 0.05 (0.01e-1)  0.20 (0.04)  -0.01e-3 (0.02e-1) | 564 | 0.59*** | 0.84*** | 0.39 | 0.20 |
| **SR^2^**  **(SR-SRmean)**  **SR^2^ * (SR-SRmean)** | 0.07e-6 (0.01e-5)  0.01 (0.05e-1)  0.02 (0.04e-3) | 561 | 0.52*** | 0.70*** | 0.39 | 0.20 |
| **RH**  **(RH-RHmean)**  **(RH)*(RH-RHmean)** | 0.02 (0.01)  0.10 (0.08)  -0.02e-1 (0.01e-1) | 613 | 0.40* | 0.50*** | 0.19 | 0.47 |
| **P^2^**  **(P^2^ - Pmean)**  **P^2^ * (P ^2^-Pmean)** | 0.04e-04 (0.04e-4)  0.05e-1 (0.02e-1)  -0.04e-6 (0.02e-6) | 603 | 0.40* | 0.40* | 0.39 | 0.42 |
| **Expected Values**  **i.e. null distribution**  **(95% CI)** |  |  | 0.25  (0.16 0.34) | 0.25  (0.14 0.38) | 0.24  (0.09 0.42) | 0.25  (0.00 0.53) |

p < 0.05, ** p < 0.01, *** p < 0.001

Table S2: Same as Table S1 but analysis was limited to primary peaks (n=76).

|  |  |  | **Proportion of peaks accurately predicted by each model** | | | |
| --- | --- | --- | --- | --- | --- | --- |
| **Climatologies** | **Coefficients** | **AIC** | **All Peaks**  **n = 76** | **High Latit.**  **n = 47** | **Middle Latit.**  **n = 20** | **Low Latit.**  **n = 9** |
| **T**  **(T - Tmean)**  **T* (T -Tmean)** | 0.04 (0.02e-1)  0.21 (0.05)  0.01e-1 (0.02e-1) | 466 | 0.65*** | 0.85*** | 0.35 | 0.33 |
| **SH^2^**  **(SH^2^ - SH^2^mean)**  **SH^2^ * (SH^2 -^ SH^2^mean)** | 0.01e-1 (0.01e-1)  0.49 (0.07)  -0.01e-1 (0.03e-2) | 477 | 0.62*** | 0.81*** | 0.35 | 0.22 |
| **SR**  **(SR-SRmean)**  **SR* (SR-SRmean)** | 0.01e-1 (0.05e-1)  0.01 (0.01)  0.04e-3 (0.04e-3) | 454 | 0.60*** | 0.72*** | 0.39 | 0.33 |
| **RH**  **RH-RHmean**  **(RH)*(RH-RHmean)** | 0.02 (0.01)  0.10 (0.08)  -0.02e-1 (0.01e-1) | 516 | 0.43*** | 0.53*** | 0.10 | 0.67** |
| **P^2^**  **(P^2^ - Pmean)**  **P^2^ * (P ^2^-Pmean)** | 0.04e-04 (0.09e-4)  0.02e-1 (0.02e-1)  -0.05e-7 (0.02e-6) | 528 | 0.37* | 0.43* | 0.35 | 0.22 |
| **Expected Values**  **i.e. null distribution**  **(95% CI)** |  |  | 0.24  (0.13 0.38) | 0.24  (0.16 0.36) | 0.20  (0.05 0.45) | 0.20  (0.00 0.55) |

* p < 0.05, ** p < 0.01, *** p < 0.001

**Figure S1.** **Comparison of climate datasets.** (A) Comparison of monthly values for the GR_cl_ and the GR_mtchd_ derived datasets (see Text S3 for details). These plots suggest that using monthly climate values from the periods that correspond to the epidemiological study periods used to predict each influenza peak versus a climatology of an arbitrary period does not have a large effect on the monthly values. (B) Comparison of monthly values for the GR_cl_ and the CRU datasets indicating that differences in spatial resolution across datasets generates substantial differences between monthly values across sites.
